# Influence of height on endothelial maintenance activity: a narrative review

**DOI:** 10.1186/s12199-021-00941-5

**Published:** 2021-02-06

**Authors:** Yuji Shimizu, Takahiro Maeda

**Affiliations:** 1 Department of Community Medicine, Nagasaki University Graduate School of Biomedical Sciences, Nagasaki-shi, Sakamoto 1-12-4, Nagasaki, 852-8523 Japan; 2 Department of Cardiovascular Disease Prevention, Osaka Center for Cancer and Cardiovascular Diseases Prevention, Osaka, Japan; 3 Department of General Medicine, Nagasaki University Graduate School of Biomedical Sciences, Nagasaki, Japan

**Keywords:** Aging, Atherosclerosis, Bone marrow, BMI, BRAP, CD34, Height, Hypertension, Muscle, Stroke

## Abstract

Recent studies have revealed an inverse association between height and cardiovascular disease. However, the background mechanism of this association has not yet been clarified. Height has also been reported to be positively associated with cancer. Therefore, well-known cardiovascular risk factors, such as increased oxidative stress and chronic inflammation, are not the best explanations for this inverse association because these risk factors are also related to cancer. However, impaired blood flow is the main pathological problem in cardiovascular disease, while glowing feeding vessels (angiogenesis) are the main characteristic of cancer pathologies. Therefore, endothelial maintenance activity, especially for the productivity of hematopoietic stem cells such as CD34-positive cells, could be associated with the height of an individual because this cell contributes not only to the progression of atherosclerosis but also to the development of angiogenesis. In addition, recent studies have also revealed a close connection between bone marrow activity and endothelial maintenance; bone marrow-derived hematopoietic stem cells contribute towards endothelial maintenance. Since the absolute volume of bone marrow is positively associated with height, height could influence endothelial maintenance activity. Based on these hypotheses, we performed several studies. The aim of this review is not only to discuss the association between height and bone marrow activity, but also to describe the potential mechanism underlying endothelial maintenance. In addition, this review also aims to explain some of the reasons that implicate hypertension as a major risk factor for stroke among the Japanese population. The review also aims to clarify the anthropological reasons behind the high risk of atherosclerosis progression in Japanese individuals with acquired genetic characteristics.

## Introduction

Recently, height has been reported to be inversely associated with hypertension [1, 2] and cardiovascular disease [3–7], while it is positively associated with cancer [3, 8]. However, a background mechanism that could explain these associations has not yet been discovered.

Age-related impairment of blood flow induces hypoxia, which increases oxidative stress [9] and activates tissue inflammation [10]. Since oxidative stress and inflammation are well-known cardiovascular risk factors [11], these could play a significant role in deciphering the association between height and cardiovascular disease.

However, oxidative stress and inflammation are also important contributors to the development and progression of cancer [12, 13]. Therefore, oxidative stress and inflammation could not be the main reason for these opposite associations, such that height is inversely associated with cardiovascular disease and positively associated with cancer.

Since impaired blood flow is the main pathological problem in cardiovascular disease while glowing feeding vessels (angiogenesis) are the main pathological conditions in cancer [14], we focused on endothelial maintenance capacity.

Recent studies revealed a close connection between endothelial maintenance activity and bone marrow activity; bone-derived hematopoietic stem cells play an important role in endothelia repair [15, 16]. However, hematopoietic bone marrow activity declines with age [17, 18]. Since the absolute volume of bone marrow is positively associated with height, the influence of age-related decline on bone marrow could be severe in participants with short stature than in those with tall stature. Based on this hypothesis, we conducted several studies by focusing on endothelial maintenance related factors including physical status, bone marrow activity, and polymorphism, in order to construct a complicated network.

To describe the current knowledge of the aforementioned complicated network, in this review, we have discussed the following: (1) association among hypertension, atherosclerosis, oxidative stress, and bone marrow activity; (2) influence of height on the association among hypertension, atherosclerosis, oxidative stress, and bone marrow activity; (3) clinical characteristics categorized by short stature and body mass index (BMI); (4) epidemiological trend of incidences of hypertension, stroke, and myocardial infarction among Japanese individuals by focusing on height and BMI status; (5) genetic characteristics that support our hypothesis; (6) the association among muscle strength, hypertension, and atherosclerosis; (7) summary of potential mechanism underlying endothelial maintenance; and (8) perspective of present study.

### Field of our study

To perform an efficient research on the influence of aging on health conditions, a pertinent issue in Japan, a high-aging field as well as a comparatively low-aging field is necessary. Primarily, we used two study fields, Goto city and Saza town, both of which belong to Nagasaki prefecture, located in the western part of Japan. In addition to the annual health check-up recommended by the Japanese government, we conducted our own surveys.

### Goto city

Remote medical care is necessary in Goto city. For providing daily clinical support, serving the bases of education and research on remote islands, and progressing the inter-professional education, we established an Island Medical Research Institute in Goto Central Hospital in 2004. As a part of this project, a Goto city field-based study has been started. To develop cooperation among the University, Nagasaki Prefectural Government, and Goto City Office, we established the Institute of Preventive Medicine in 2013. In addition, in 2010, the Department of Dentistry also joined our project and established the Island Dental Health Research Institute. This facility is aimed at providing dental care in rural areas, serving the bases of education and research on remote islands, and progressing the inter-professional education. Furthermore, in 2012, as the Regional Headquarters of Community-based Medical Education, which supports the medical students of the regional quota, we established the Department of Community Medicine, Nagasaki University Graduate School of Biomedical Science. This facility is also aimed at enhancing the research on community medicine and community-based medical education. Therefore, several facilities are involved in our consortium and facilitating progress in research, education, and daily medical services. The rate of aging in Goto city is high. According to an estimation by the National Institute of Population and Social Security Research in 2013, the total number of residents aged 40 years or older in 2015 was 27,206, and among them, the total number of residents aged ≥ 65 years was 14,018 (51.5%) [19].

Owing to the shortage of staff to conduct a health check-up in the present survey, the entire city could not be surveyed in 1 year. Therefore, we conducted the survey in different parts of the city over a period of 3 years to ensure that all the areas of Goto city were covered. In our survey, we evaluated arterial stiffness using two methods: carotid intima-media thickness (CIMT) and cardio-ankle vascular index (CAVI). We also evaluated muscle strength using handgrip strength and tongue pressure. In addition, we conducted an oral survey in this field.

### Saza town

Saza town is located on the main island of Kyusyu. Unlike Goto city, Saza town is known as a bed town adjacent to the Sasebo city, where the total number of residents aged 40 years or above was 156,771 in 2015 [19]. Residents of Saza town are comparatively younger than those in Goto city. According to an estimation by the National Institute of Population and Social Security Research in 2013, the total number of residents aged 40 years or older in 2015 was 7883, and among them, the total number of residents aged ≥ 65 years was 3498 (44.4%) [19]. A Saza town-based study was started in 2014. Unlike the survey in Goto city, we conducted an annual survey every year and evaluated arterial stiffness using CIMT. We also conducted a thyroid survey using ultrasonography.

Both fields had stocks of blood samples. However, in the present condition, genetic surveys were only approved in Goto city because the informed consent that was obtained did not include the genetic information from Saza town.

## Data collection

Our study is the largest general population-based prospective study in the world that deals with circulating CD34-positive cells in a strict manner by limiting the target population of men within a narrow range of age.

In addition, we evaluated the organic (structual) value of arterial stiffness and functional values of arterial stiffness in an accurate manner. These are the strengths of our study.

### Measurement of CD34-positive cell count

A heparin sodium tube was used to count the number of CD34-positive cells. To measure CD34-positive cell counts, an automated software on the BD (Beckton Dickinson Biosciences) FACSCant^TM^ II system was used in accordance with the International Society of Hematotherapy and Graft Engineering (ISHAGE) guidelines [20]. Measurement of CD34-positive cells required fresh samples (within 24 h after blood collection). Approximately 30 min was required to measure one sample. Since the maximum number of samples to measure CD34-positive cells in a day is limited to 20 samples, we limited the measurement of CD34-positive cells among men aged 60–69 years who participated in a general health check-up in Goto city and Saza town.

### Measurement of carotid intima-media thickness (CIMT)

An experienced vascular examiner measured both left- and right-sided CIMT of the common carotid arteries using LOGIQ Book XP with a 10-MHz transducer (GE Healthcare, Milwaukee, WI, USA). Maximum values for the left and right common CIMT were then calculated using automated digital edge-detection software (Intimascope; MediaCross, Tokyo, Japan), using a previously described protocol [21]. The recently developed Intimascope software was used to increase the accuracy and reproducibility of CIMT measurement values. Semi-automatically, this software recognizes the edges of the internal and external membranes of the artery and automatically determines the distance at a sub-pixel level (estimated to be 0.01 mm) [22]. In this way, we accurately evaluated the organic values of arterial stiffness.

### Measurement of cardio-ankle vascular index (CAVI)

Brachial-ankle pulse wave velocity (PWV) measurements are generally used to evaluate functional values of arterial stiffness. Since PWV measurements can be strongly affected by blood pressure [23], CAVI was recently developed in Japan for these measurements to avoid the confounding effects of blood pressure [24]. In the current study, CAVI was determined using a Vasera VS-1000 vascular screening system (Fukuda Denshi, Tokyo, Japan), with the participant resting in a supine position. The underlying principles of CAVI have been described elsewhere by Yambe et al. [25]. In this way, we accurately evaluated the functional values of arterial stiffness.

## Results

In line with the topics discussed in this article, we performed several studies using the survey data from Goto city and Saza town. The parts of those studies are as follows.

### Active arterial wall thickening, baseline atherosclerosis, and CD34-positive cells [26]

Based on the data derived from 363 men aged 60–69 years, a 2-year follow-up study was performed. In this study, active arterial wall thickening [CIMT progression (≥ 0.01 mm/year)] inversely associated with baseline atherosclerosis (CIMT ≥ 1.1 mm); the fully adjusted odds ratios (ORs) of active arterial wall thickening for baseline atherosclerosis was 0.24 (0.11, 0.52). This study also showed significant positive association between active arterial wall thickening and CD34-positive cells among subjects without hypertension. The cardiovascular risk factor-adjusted ORs of active arterial wall thickening for the logarithmic circulating CD34+ cell count were 0.69 (0.36, 1.32) for subjects with hypertension and 1.83 (1.19, 2.84) for subjects without hypertension.

### Gamma-glutamyl transpeptidase (γ-GTP), hypertension, and atherosclerosis in relation to CD34-positive cells [27] (Fig. 1)

In the cross-sectional study based on 562 elderly men aged 60–69 years, among participants with high CD34-positive cells, γ-GTP showed significant and positive association with atherosclerosis, but not with hypertension. With the reference group of the lowest quartile of γ-GTP level, the cardiovascular risk factor-adjusted ORs for the highest quartile of γ-GTP level were 4.28 (1.34, 13.63) for atherosclerosis and 1.02 (0.46, 2.28) for hypertension. Among participants with low CD34-positive cells, γ-GTP showed no significant association with atherosclerosis [OR = 0.61 (0.21, 1.81)], but was significantly and positively associated with hypertension [OR = 2.84 (1.30, 6.22)].

**Fig. 1:**
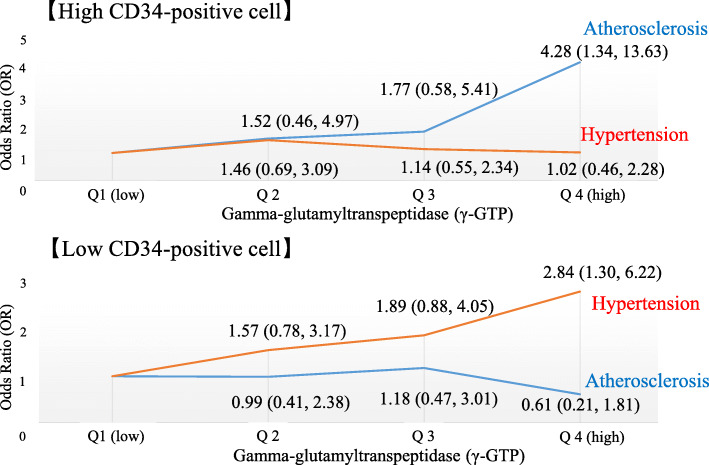
Associations among γ-GTP, hypertension, and atherosclerosis based on levels of CD34-positive cells [27]

### Cardio-ankle vascular index (CAVI), carotid intima-media thickness (CIMT), and CD34-positive cell [28] (Fig. 2)

A cross-sectional study was performed on 249 elderly Japanese men aged 60–69 years. For subjects with low circulating CD34-positive cell levels, logarithmic values of circulating CD34-positive cells were inversely associated with CAVI (cardiovascular risk factor adjusted multivariable standardized parameter estimate (*β*) = − 0.22, *p* = 0.014) but not for subjects with high levels (*β* = − 0.04, *p* = 0.638). In addition, even when no significant associations between CAVI and CIMT were detected in the subjects with low circulating CD34-positive cell levels (*β* = − 0.02, *p* = 0.865), significant positive associations were identified in subjects with high levels (*β* = 0.22, *p* = 0.028).

**Fig. 2:**
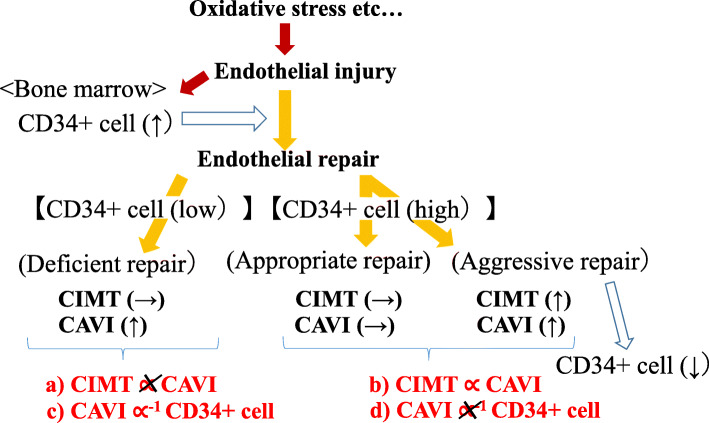
Functional arterial stiffness and organic arterial stiffness in relation to circulating CD34-positive cells [28]. CAVI, cardio-ankle vascular stiffness index; CIMT, carotid intima-media thickness; CD34+, CD34-positive

### Height and CD34-positive cells in relation to CD34-positive cell levels [29]

A cross-sectional study was conducted on 231 elderly Japanese men aged 65–69 years. No significant correlation between height and age was observed with a simple correlation coefficient of (*r*) = − 0.12 (*p* = 0.08). In participants with low levels of circulating CD34-positive cells, height was significantly associated with circulating CD34-positive cells but not for those with higher levels, with a cardiovascular risk factor-adjusted *β* of 0.27 (*p* = 0.008) for low and 0.11 (0.275) for higher circulating CD34-positive cell levels.

### Height and atherosclerosis in relation to body mass index (BMI) [30]

A cross-sectional study based on 1337 men aged 30–89 years revealed that no significant association between height and atherosclerosis (CIMT ≥ 1.1 mm) was observed for non-overweight individuals [body mass index (BMI) < 25 kg/m^2^], whereas a significant inverse association was observed between the two in overweight individuals (BMI ≥ 25 kg/m^2^). The known cardiovascular risk factor-adjusted OR of atherosclerosis for an increment of one standard deviation in height (6.70 cm) was 1.05 (0.87, 1.27) for non-overweight and 0.71 (0.54, 0.94) for overweight individuals.

### BRAP (rs3782886), aldehyde dehydrogenase 2: ALDH2 (rs671), and hypertension [31]

To determine the mechanism underlying the single nucleotide polymorphism in breast cancer suppressor BRCA1-related associated protein (BRAP, rs3782886) and aldehyde dehydrogenase 2 (ALDH2, rs671) and hypertension, a multi-faceted analysis was performed in a simple general elderly population model (1313 older Japanese aged 60–98 years). These minor alleles of the genetic factors (rs3782886 and rs671) were found to be inversely associated with hypertension and positively associated with platelet count only in individuals with high levels of reticulocytes. Further, these minor alleles (rs3782886 and rs671) were found to be positively associated with never-drinkers. Moreover, only in individuals with high reticulocyte levels, never-drinkers showed significantly higher platelet counts than non-never-drinkers.

### Handgrip strength and organic values of atherosclerosis in relation to platelet levels [32]

A cross-sectional study based on 795 elderly hypertensive Japanese subjects aged 60–89 years showed that no significant association between handgrip strength and organic values of atherosclerosis (CIMT ≥ 1.1 m) was observed for subjects with lower platelet counts, whereas a significant positive association was observed for subjects with higher platelets; adjusted ORs and 95% CIs of organic values of atherosclerosis for 1 SD increments in handgrip strength were 0.86 (0.61, 1.22) for subjects with lower platelets and 1.82 (1.26, 2.64) for subjects with higher platelets.

## Discussions

### Atherosclerosis and active arterial wall thickening

Even increased CIMT, a well-known surrogate marker of atherosclerosis [33], is an established risk factor for cardiovascular disease [34], no association between yearly CIMT progression and cardiovascular disease has been reported [35]. In our previous study, active arterial wall thickening, which is defined as yearly CIMT progression (≥ 0.01 mm), is inversely associated with baseline atherosclerosis (CIMT ≥ 1.1 mm) [26]. Therefore, participants with established atherosclerosis should have a lower chance of active arterial wall thickening (yearly CIMT progression). Since atherosclerosis is a result of aggressive endothelial repair while active arterial wall thickening is due to an aggressive endothelial repair, deficiency of endothelial repair activity could blame this paradoxical phenomenon.

### Active arterial wall thickening and CD34-positive cell

Bone marrow-derived hematopoietic stem cells (CD34-positive cells) contribute to endothelial repair [15] not only by differentiation into endothelial cells but also into foam cells, which are known sources of atherosclerosis [16]. Therefore, the presence of CD34-positive cells is necessary for active arterial wall thickening. In fact, active arterial wall thickening (CIMT ≥ 0.01 mm/year) is positively associated with circulating CD34-positive cells [26]. However, this association was limited to participants without hypertension.

### Hypertension and reduced circulating CD34-positive cell

Hypertension could act as a strong confounding factor in the association between circulating CD34-positive cells and active arterial wall thickening [26]. Hypertension strongly injures the endothelium. Under such conditions, endothelial repair that stimulates CD34-positive cell production should be activated. However, reduction of these cells could also be observed due to other pathways; for instance, many of these cells differentiated into mature cells (CD34-negative cells). Therefore, hypertension could act as a strong confounding factor in the association between circulating CD34-positive cells and endothelial repair activity, including active arterial wall thickening [26, 36, 37].

### Hypertension and atherosclerosis in relation to circulating CD34-positive cell levels

CD34-positive cells are also known to contribute to angiogenesis [38, 39], which plays an important role in the maintenance of microcirculation by increasing blood flow. Since ischemic conditions are known to be associated with oxidative stress, angiogenesis can reduce the magnitude of oxidative stress. Oxidative stress has been reported to play a crucial role in the pathogenesis of atherosclerosis [40] and hypertension [41]. Since circulating CD34-positive cells are necessary for active arterial wall thickening (CIMT ≥ 0.01 mm/year) [26], participants with high circulating CD34-positive cells might have higher activity of progression of atherosclerosis and angiogenesis. In other words, among participants with high levels of circulating CD34-positive cells, the presence of atherosclerosis also indicates the presence of angiogenesis, which reduces the risk of hypertension by decreasing oxidative stress.

Serum concentration of gamma-glutamyl transpeptidase (γ-GTP) could act as a marker of oxidative stress [42, 43]. As aging is also known to be associated with oxidative stress [44, 45], γ-GTP could act as a marker of risk factors for age-related diseases such as atherosclerosis and hypertension in elderly subjects. Our previous cross-sectional study indicated that active endothelial repair associated with atherosclerosis could have a preventive effect on the development of hypertension. Among participants with high circulating CD34-positive cells (≥ median values), γ-GTP showed a positive association with atherosclerosis but not with hypertension. However, among participants with low circulating CD34-positive cells (< median values), γ-GTP showed no significant association with atherosclerosis, although there was a positive association with hypertension (Fig. 1) [27]. This study indicates that even increased oxidative stress induces progression of atherosclerosis, and hypertension could not be observed among participants with sufficient capacity to activate endothelial repair. Therefore, active endothelial repair associated with atherosclerosis could prevent hypertension to the same extent.

**Fig. 3:**
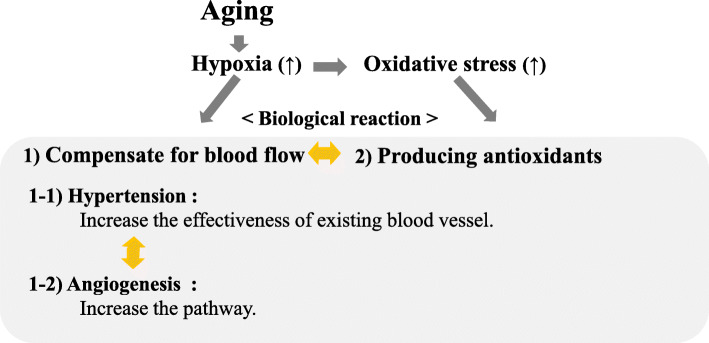
Potential biological reaction to oxidative stress. Aging increases hypoxia and oxidative stress. To adjust for hypoxia, stimulating a compensation for blood flow (hypertension and angiogenesis) and producing antioxidants are programmed in living beings

Aging is a process that increases hypoxia, which contributes to functional decline [46]. Hypoxia is also known to be associated with oxidative stress [47, 48]. Therefore, not only producing antioxidants but also stimulating a compensation for blood flow is programmed for living creature to adjust for hypoxia (Fig. 3). There are mainly two ways to compensate blood flow the first one is hypertension and next one is angiogenesis. Hypertension is a phenomenon that is intended to increase blood flow by increasing the effectiveness of the present vascular system. Angiogenesis also increases blood flow by creating new vessels.

Therefore, three factors (hypertension, angiogenesis, and antioxidants) can maintain balance. If the influence of angiogenesis and anti-oxidative effect is appropriate, hypertension is not necessary to adjust for oxidative stress; however, if the influence of angiogenesis and/or anti-oxidative effect is not enough, hypertension is necessary. Further investigation of this topic is necessary.

### Functional arterial stiffness and organic arterial stiffness in relation to circulating CD34-positive cell levels

Even participants with high circulating CD34-positive cells are associated with active endothelial repair [26, 27, 36, 37], and the influence of endothelial deficiency on the endothelium has not yet been clarified.

CAVI reflects arteriosclerosis of the aorta, arterial distensibility [49], and change in contractility of smooth muscle cells [24]. Therefore, CAVI reflects functional arterial stiffness.

On the other hand, measurement of CIMT using ultrasonography of the left and right carotid arteries, which were calculated using automated digital edge-detection software (Intimascope; MediaCross, Tokyo, Japan) [21, 22] indicates organic arterial stiffness.

To evaluate the influence of endothelial repair activity on the association between organic values of arterial stiffness and functional values of arterial stiffness, we conducted further analyses. In this study, we evaluated the association between the organic value of arterial stiffness and functional arterial stiffness in relation to the activity of endothelia repair evaluated by circulating CD34-positive cells [28]. We found that even when no significant associations between CAVI and CIMT were detected in subjects with low circulating CD34-positive cell levels (median <) (Fig. 2(a)), significant positive associations were identified in subjects with high levels (Fig. 2(b)). In addition, for subjects with low circulating CD34-positive cell levels (median <), circulating CD34-positive cells were inversely associated with CAVI (Fig. 2(c)) but not in subjects with high levels (≥ median) (Fig. 2(d)).

Therefore, only among participants with sufficient circulating CD34-positive cells (≥ median), organic values of arterial stiffness indicate functional values of arterial stiffness. In addition, only among participants with low circulating CD34-positive cells, deficiency of CD34-positive cells indicates functional values of arterial stiffness. Since CD34-positive cells not only contribute to the development of organic values of arterial stiffness [26] but also to the progression of angiogenesis [38, 39], the sensitivity of the impaired blood flow should be much stronger for functional values of arterial stiffness than that of organic values of arterial stiffness. These studies indicate that not having organic values of atherosclerosis does not always indicate beneficial conditions for blood flow, and circulating CD34-positive cell levels play an important role in the regulation of the circulation system.

### Chronic kidney disease (CKD) and atherosclerosis in relation to circulating CD34-positive cell

Since bone marrow-derived hematopoietic stem cells (CD34-positive cells) contribute to endothelial repair [15], bone marrow activity is crucial for the progression of atherosclerosis. Reduced renal function is known to be associated with reduced bone marrow activity, namely renal anemia. However, reduced renal function such as CKD has been reported to be positively associated with atherosclerosis [50]. On further analysis, CKD is revealed to be positively associated with CIMT only in participants with high circulating CD34-positive cells (≥ median value), whose productivity of CD34-positive cells is comparatively high [51]. Circulating CD34-positive levels may also act as a determinant factor in the association between CKD and CIMT.

### Height and bone marrow activity (circulating CD34-positive cell)

These studies indicate that the productivity of CD34-positive cells plays an important role in the maintenance of the endothelium. However, the factor that determines the capacity of CD34-positive cell production is not yet known. It is known that hematopoietic bone marrow activity declines with age [17, 18], which might induce a lower capacity for endothelial maintenance in elderly subjects. Adult height is positively associated with the absolute volume of bone marrow. We hypothesized that adult height may act as a determinant factor in the capacity for CD34-positive cell production. Since analysis among participants with low circulating CD34-positive cells (< median) strengthens their influence, which results in a deficiency of endothelial repair [28], the positive association between height and circulating CD34-positive cells is limited to participants with low circulating CD34-positive cells (< median), as shown in our previous study [29]. Progression of cancer requires angiogenesis, and CD34-positive cells play an important role in this process [38, 39]. Therefore, the fact that there is a positive association between height and cancer [3, 8] can also be explained by this mechanism: height determines the level of CD34-positive cell production.

### Height and bone marrow activity (reticulocyte)

Reticulocytes, which are immature cells of red blood cells, also indicate hematopoiesis. Therefore, reticulocyte levels could also be positively associated with height. We then performed further analysis showing that height is positively associated with reticulocytes in elderly men, particularly in men with a high hemoglobin concentration (≥ 14.5 g/dL )[52]. Since lower productivity of reticulocytes results in anemia, we also evaluated the association between height and anemia (hemoglobin < 13.0 g/dL) among the general Japanese men and found that height is inversely associated with anemia, and the status of drinking is likely to be confounded by this association [53]. It has been reported that hemoglobin levels of drinkers are higher than those of non-drinkers [54, 55]; drinkers in our study showed slightly but significantly higher hemoglobin levels than non-drinkers [53]. Therefore, drinking may accelerate hemoglobin production.

### Reticulocyte and hypertension, and atherosclerosis

Unlike CD34-positive cells, which directly contribute to endothelial repair [15, 16], reticulocytes also contribute to endothelial maintenance by reducing oxidative stress, which plays a crucial role in the pathogenesis of both atherosclerosis [40] and hypertension [41]. We found that reticulocyte levels were positively associated with hypertension and inversely associated with atherosclerosis (CIMT ≥ 1.1 mm) [56]. Therefore, we hypothesized that oxidative stress induces hypertension and stimulates reticulocyte production. Increased levels of reticulocytes could reduce the magnitude of endothelial injury by reducing oxidative stress.

### Hemoglobin and hypertension, and atherosclerosis

Increase in the level of oxidative stress elevates the concentration of hemoglobin. Therefore, hemoglobin is also found to be positively associated with hypertension [57, 58] and hypertension-associated vascular damage, which is evaluated by hepatocyte growth factor (HGF) [59]. CAVI indicates functional values of arterial stiffness and the manufacturer’s recommendation defined CAVI ≥ 9 as an abnormal [60]. Next, we defined functional values of atherosclerosis as CAVI ≥ 9 and observed a positive association between hemoglobin and functional values of atherosclerosis based on CAVI score [61].

### Salt-sensitive hypertension

Salt intake was reported to be positively associated with hypertension [62]. Endothelial dysfunction, renin-angiotensin aldosterone system, ion transport, and decrease in estrogen levels are known components that contribute to salt-sensitive hypertension [63]. Endothelial dysfunction damages the mechanism of upregulating nitric oxide (NO) production. Since NO plays an important role in reducing blood pressure by dilating blood vessels [64], endothelial dysfunction is more frequent in salt-sensitive than in salt-resistant essential hypertension [65]. Therefore, the capacity of endothelial repair associated with height could influence salt sensitivity. Further investigation of this topic is necessary.

### Height and hypertension

Previous studies have shown an inverse association between height and hypertension [1, 2]. Increased levels of oxidative stress evaluated by γ-GTP are positively associated with hypertension among elderly participants with endothelial repair deficiency, which is associated with lower levels of circulating CD34-positive cells [27]. The capacity to reduce oxidative stress could be positively associated with height because height is influenced by the capacity of reticulocyte production [52, 53]. The capacity of endothelial repair (productivity of CD34-positive cells) is also positively associated with height [29]. Therefore, height could be inversely associated with hypertension since it acts as an indicator of the capacity to reduce oxidative stress and the ability of endothelial repair. Furthermore, the capacity of endothelial repair could influence salt sensitivity, as mentioned above, and height could be associated with salt-sensitive hypertension. Further investigation in this regard is necessary.

### Height and atherosclerosis

The capacity to reduce oxidative stress evaluated by the productivity of reticulocytes and hemoglobin could be positively associated with height [52, 53]. Oxidative stress has been reported to play a crucial role in the pathogenesis of atherosclerosis [40] and hypertension [41]. Previous studies have shown an inverse association between height and hypertension [1, 2]. Height could be inversely associated with atherosclerosis (increased CIMT). However, atherosclerosis is a result of aggressive endothelial repair. Therefore, the inverse association between height and atherosclerosis could only be observed in participants with elevated oxidative stress. Since BMI is positively associated with oxidative stress [66], the inverse association between height and atherosclerosis is found to be limited to overweight individuals (BMI ≥ 25 kg/m^2^) [30]. However, hematopoietic bone marrow activity declines with age [17, 18], and a positive association between height and circulating CD34-positive cells was observed only in participants with low circulating CD34-positive cells (< median) [29]. Since CD34-positive cells are necessary to increase CIMT [26], while height determines the capacity of CD34-positive cell production [29] under the influence of age-related decline in bone marrow activity [17, 18], aging may reduce the likelihood of progressing atherosclerosis (increased CIMT), especially in short-statured individuals. In such a condition, to compensate for age-related oxidative stress [44, 45], hypertension plays an important role [27]. In our previous study, we found that the status of hypertension determines the association between height and CD34-positive cells; a positive association between these factors was observed only in men with hypertension [67]. This study also supports the abovementioned mechanism that aging may reduce the chance of progressing atherosclerosis (increased CIMT), especially for short-statured individuals because the presence of hypertension among the elderly enhances the influence on insufficient endothelial repair due to a shortage of CD34-positive cells [27]. A significant positive association between height and CD34-positive cells was observed among hypertensive subjects [67].

Under such conditions, even if the chance of progressing atherosclerosis (increased levels of CIMT) is reduced, the risk of atherosclerotic disease becomes high [35] due to the lack of endothelial repair, including angiogenesis.

### Height and stroke

Many recent studies have reported an inverse association between height and cardiovascular disease, including stroke incidence [3–7]. Since an inverse association between height and atherosclerosis was observed only in Japanese participants who were overweight (BMI ≥ 25 kg/m^2^) [30], we performed another analysis stratified by BMI status. We found that the inverse association between height and incidence of stroke was observed only in Japanese participants without high BMI (BMI < 23 kg/m^2^) [7]. Therefore, the risk of stroke in short-statured individuals could not be explained by the presence of atherosclerosis.

Short stature and lower BMI are well-known characteristics of Japanese. Hypertension is a major risk factor for stroke in the Japanese population [68].

Active endothelial repair that is associated with atherosclerosis may prevent hypertension to the same extent [27], possibly by activating angiogenesis, which increases blood flow in peripheral tissues. Circulating CD34-positive cells are necessary to activate endothelial repair [26], while atherosclerosis is the result of aggressive endothelial repair. In addition, the positive association between height and circulating CD34-positive cells was observed to be limited to participants with low circulating CD34-positive cells (< median), as shown in our previous study [29]. Therefore, the risk of stroke incidence in patients with short stature could be explained by the deficiency of circulating CD34-positive cells, which indicates a deficiency of endothelial repair.

Small artery dysfunction known as arteriosclerosis causes hemorrhagic stroke and lacunar infarction, while large artery dysfunction known as atherosclerosis causes large artery occlusive infraction. Since hemorrhagic stroke and lacunar infarction, but not large artery occlusive infarction, are the major subtypes of stroke in Japanese [68], deficiency of endothelial repair could play an important role in the incidence of stroke among Japanese.

Previous studies have shown an inverse association between height and hypertension [1, 2]. In our previous study, circulating CD34-positive cells were found to be positively correlated with height limited to participants with hypertension [67]. These studies also support the abovementioned mechanism because, even though hypertension stimulates CD34-positive cell production, height determines the maximum productivity of CD34-positive cells.

Previously, BMI was reported to be substantially lower in Japanese men living in Japan than in those living in California and Hawaii [69]. The 20-year follow-up prospective study with the Honolulu Heart Program revealed that BMI is not associated with stroke among Japanese-American men in Hawaii [70]. Furthermore, a previous autopsy study reported that Japanese individuals living in Honolulu had significantly more atherosclerosis of the circle of Willis, but less intra-parenchymal artery sclerosis and less cerebral infarction than those living in Japan [71]. CD34-positive cells maintain the structural integrity of intra-parenchymal blood vessels [72], while height is positively associated with circulating CD34-positive cells [29, 67]. In addition, a previous study with a history of atherothrombotic cerebral ischemic events reported a strong inverse association between circulating CD34-positive cells and the number of cerebral infarcts. However, this study did not find any correlation between the degrees of atherosclerosis and circulating CD34-positive cells [73]. These studies also support our hypothesis that the deficiency of endothelial repair induces the risk of stroke in Japanese.

### Clinical characteristics categorized by status of short stature and high BMI

**Fig. 4:**
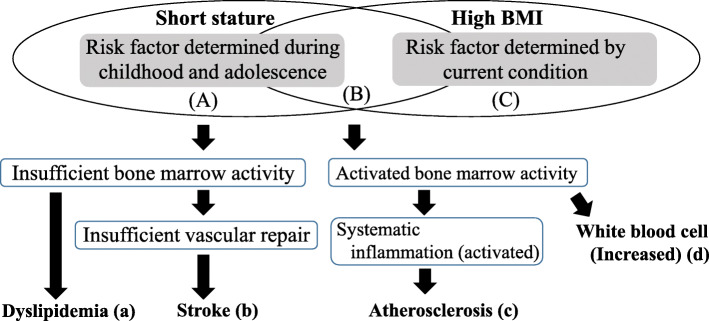
Association between short stature and high BMI on bone marrow activity [74]. (**A**) Short stature without a high BMI. (**B**) Short stature with a high BMI. (**C**) High BMI without short stature. A higher risk of stroke was observed among (**A**) [7], while a higher prevalence of atherosclerosis was observed among (**B**) [30]

Figure 4 shows the clinical characteristics categorized by short stature and high BMI.

Height is regarded as a marker for social and physical conditions during childhood (adolescence), including genetic factors [4, 5, 7, 30, 74], while high BMI, which is reported to be positively associated with increased risk of disease [75], is largely influenced by current conditions. Therefore, the first model that is composed of those with a short stature but not a high BMI was designed to elucidate the potential effect of childhood (adolescent) as cardiovascular risk (Fig. 4(A)). The second model, with a short stature and a high BMI, reflecting both childhood (adolescent) and current risk features combined cardiovascular risk (Fig. 4(B)), and the third model with a high BMI but not a short stature was designed to elucidate the potential effect of the current condition (Fig. 4(C)). The details of this concept have been described elsewhere [74].

A previous study revealed a significant inverse association between height and stroke in participants with a BMI < 23 kg/m^2^ but not with a BMI ≥ 23 kg/m^2^ [7] (Fig. 4(b)). Another study showed that height was inversely associated with dyslipidemia in participants with a BMI < 25 kg/m^2^ but not with a BMI ≥ 25 kg/m^2^ [74] (Fig. 4(a)).

Moreover, the inverse association between height and activated inflammation evaluated by high white blood cell count (highest tertiles of white blood cell count) was limited to participants with a BMI ≥ 23 kg/m^2^ [76] (Fig. 4(d)). There was also an inverse association between height and carotid atherosclerosis, which was limited to participants with a BMI ≥ 25 kg/m^2^ [30] (Fig. 4(c))

Therefore, activated inflammation and progression of atherosclerosis could not be established only by short stature; however, dyslipidemia and the incidence of stroke could be established without inflammation. Further investigation is required to understand the influence of height and high BMI on the incidence of coronary heart disease.

### Trend of hypertension and incidence of stroke among Japanese

In 1960, the population-wide screening of hypertension began in Japan. A national survey conducted between 1971 and 1990 detected decreased systolic blood pressure [77]. This phenomenon could not be explained merely by the widespread use of antihypertensive medication [78]. The age-adjusted prevalence of hypertension, which was defined as systolic blood pressure ≥ 140 mmHg and/or diastolic blood pressure ≥ 90 mmHg and/or antihypertensive medication, increased during the earlier period from 1961 to 1983, and then decreased from 1983 to 2002 [79]. This means that blood pressure decreased despite unfavorable trends of higher prevalence of overweight (BMI > 25 kg/m^2^) (excluding young women) observed between 1980 and 2000 [80]. In addition, Japanese nationwide research in individuals aged 20 years showed an increasing trend in height during the observational period from 1892 to 1994 [81]. Short stature with a low BMI (< 23 kg/m^2^) is associated with the incidence of stroke [7]. Since an inverse association between height and hypertension has been reported [1, 2], while hypertension is a major risk factor for stroke [68], getting taller and increasing BMI during recent periods could reduce the risk of stroke among Japanese individuals. In fact, the Hisayama study reported that the age-adjusted incidence of stroke decreased greatly by 51% in men and by 43% in women from the 1960s to the 1970s [79].

Endothelial dysfunction is more frequent in salt-sensitive than in salt-resistant essential hypertension [65]. Therefore, the capacity of endothelial repair associated with height might also influence salt sensitivity. Stroke mortality, which could be attributed to high sodium intake in Japan, has shown a gradual decline from 1990 to 2016 [82]. These reports suggest that reducing salt intake and gaining height may reduce the risk of stroke among the Japanese population.

### Trend of incidence of myocardial infarction among Japanese

A Hisayama study reported that the incidence of acute myocardial infarction showed no clear secular change among participants aged < 79 years, whereas in participants aged ≥ 80 years, the incidence tended to increase from the 1960s to the 1980s and remained unchanged thereafter [79]. Even short stature is inversely associated with atherosclerosis as evaluated by CIMT, although this association is limited to participants with BMI ≥ 25 kg/m^2^ [30]. Unlike stroke, aggressive endothelial repair that results in atherosclerosis evaluated by CIMT could blame the incidence of myocardial infarction. Therefore, increasing the prevalence of obesity [80] may increase the risk of myocardial infarction, especially for short-statured individuals. The findings of the Japan National Health and Nutrition Survey from 1973 to 2016 revealed that both weight and height showed an increasing trend in the elderly (aged ≥ 65 years) [83]. This trend could have beneficial influence on preventing myocardial infarction to the same extent, possibly by reducing oxidative stress [44, 52, 53, 56, 57]. Therefore, no clear secular trend was observed for the incidence of myocardial infarction after the 1980s [79]. Further investigation of this trend is necessary.

### Platelet and circulating CD34-positive cell

Platelets have recently been shown to play a major role in inflammation as well as being an important initial activator for the development of atherosclerotic lesions [84]. Upon endothelium injury, the sub-endothelial components that activate platelets are elevated in peripheral blood [85, 86]. These activated platelets stimulate the mobilization of CD34-positive cells from the bone marrow [15, 87–89]. Therefore, both platelets and circulating CD34-positive cells are elevated upon endothelium injury. However, aggressive endothelium repair might cause reduction, especially for CD34-positive cells because the number of CD34-positive cells is much smaller than the number of platelets. Therefore, for subjects without aggressive endothelial repair, platelets show a positive correlation with circulating CD34-positive cells but not in subjects with aggressive endothelial repair [26, 28, 29, 36, 37, 90]. In other words, platelets could act as an important indicator of vascular repair activity [90].

### Height and endothelial repair (platelet, CD34-positive cell, reticulocyte)

A cross-sectional study of elderly men (65–69 years) with a normal range of BMI (18.5–24.9 kg/m^2^) showed that for participants without age-related bone marrow activity reduction (participants with high hemoglobin), platelets had a significant association with circulating CD34-positive cells and CIMT [91]. This study also showed that height is positively associated with reticulocytes and inversely associated with platelets among participants with high hemoglobin levels. Therefore, for participants without severe age-related decline in bone marrow activity, shorter stature may have lower activity of hematopoiesis and higher activity of endothelial repair than that of taller individuals. Since aging could not only reduce the chance of atherosclerosis progression but also inhibit the development of angiogenesis, short-statured individuals could easily progress to atherosclerosis in the middle-aged period, and it is difficult to develop angiogenesis in the later part of life. Therefore, short-statured participants may have a higher risk of cardiovascular disease than taller individuals. Further investigation with long-term follow-up is necessary to clarify this mechanism.

### BRAP and atherosclerosis, stroke, and hypertension

The minor allele of a single nucleotide polymorphism (SNP) (rs3782886) in breast cancer suppressor BRCA1**-**related associated protein (BRAP) is reported to be positively associated with myocardial infarction [92]. BRAP increases the risk of carotid atherosclerosis by activating inflammatory cascades [93]. Although there is a strong association between atherosclerosis and hypertension [36], the minor allele of rs3782886 is reported to be inversely associated with blood pressure (systolic and diastolic) [94]. These studies indicate that the genetic characteristic that has the risk of developing atherosclerosis could induce a lower risk of developing hypertension. This phenomenon supports the mechanism through which participants with high activity of endothelial repair due to progressing atherosclerosis could prevent hypertension [27]. Furthermore, a lack of association between BRAP and ischemic stroke has also been reported [95].

Atherosclerosis could not explain the risk of stroke in short-statured individuals [7, 30], and previous studies have shown an inverse association between height and hypertension [1, 2]. Hypertension is a major risk factor for stroke among Japanese individuals [68]. Therefore, among participants with short stature, progression of atherosclerosis could possibly prevent stroke incidence by reducing the risk of hypertension. Since short stature is a well-known characteristic of Japanese individuals, a variant of rs3782886 could have a beneficial influence on the longevity of the Japanese by preventing hypertension.

### BRAP and platelet, and hypertension

If the inverse association between minor rs3782886 and blood pressure (systolic and diastolic) [94] is caused by active endothelial repair including progression of atherosclerosis [93], minor rs3782886 could be positively associated with high platelet levels. This is related to hypertension since platelets could act as an indicator of vascular repair activity [90]. A strong positive association between atherosclerosis and hypertension has also been observed [26, 27, 36].

In a previous study, the minor allele of rs3782886 was found to be significantly associated with high platelet count, which is related to hypertension [96]. Furthermore, we also observed that bone marrow activity could influence the association between rs3782886 and hypertension [31]. Therefore, bone marrow activity plays an important role in preventing hypertension.

### Low tolerance to alcohol exposure

Low tolerance to ethanol exposure is a well-known characteristic of East Asians because of the high prevalence of the minor allele of ALDH2-related polymorphism rs671 [97]. Strong linkage disequilibrium (LD) was reported between rs3782886 and rs671 [98]. Therefore, the minor allele of rs3782886 was reported to be positively associated with never-drinkers [31, 99]. Since ethanol directly attenuates platelet activation [100], while the beneficial association of variant allele of rs3782886 is associated with higher platelet count [96], avoiding ethanol may be preferable to prevent hypertension in participants with variant allele of rs3782886 [31]. Due to the efficient influence on preventing hypertension by the variant of rs3782886, characteristics of low tolerance for alcohol exposure were also anthropologically widely spread among Japanese individuals.

### Muscle strength and hypertension

Low handgrip strength is positively associated with cardiovascular mortality [101]. The strength of handgrip is reported to be inversely correlated with CAVI, which reflects the functional value of arterial stiffness among the elderly [102]. Therefore, impairment of blood flow should be associated with reduced muscle strength.

Our previous study on elderly Japanese population found that in participants without hypertension, high platelet levels had a beneficial association with maintaining muscle strength but not in participants with hypertension [103]. For elderly non-hypertensive men, platelets were significantly positively associated with CD34-positive cells but not with CIMT, which reflects the organic values of arterial stiffness [90]. Since CD34-positive cells contribute to maintaining microcirculation partly by developing angiogenesis [38, 39], elderly non-hypertensive individuals with higher platelet levels might have a beneficial influence on maintaining muscle strength through higher activity of maintaining microcirculation, including progression of angiogenesis [103].

On the other hand, even hypertension is known to be associated with disruption of the circulatory system, higher blood pressure is associated with stronger handgrip in the oldest participants [104]. Since platelets could act as an indicator of vascular repair activity [90] and organic values of atherosclerosis is a result of endothelial repair, for hypertensive elderly subjects with high platelet levels, the presence of organic values of atherosclerosis could indicate active endothelial repair, which has a beneficial influence on maintenance of microcirculation, although not in those with low platelet levels.

In our previous study, a positive association between handgrip strength and carotid atherosclerosis (CIMT ≥ 1.1 mm) was found among hypertensive elderly subjects with higher but not lower platelet counts [32]. We also reported that tongue pressure was inversely associated with carotid atherosclerosis (CIMT ≥ 1.1 mm) in hypertensive men with low platelet but not in those with high levels [105].

Therefore, hypertension may have a beneficial influence on microcirculation, which might require active endothelial repair, including angiogenesis. Since height could be an indicator of endothelial repair, this can influence muscle strength among older individuals. Further investigation is required on this topic.

### Summary of potential mechanism underlying endothelial maintenance activity in relation to height

**Fig. 5:**
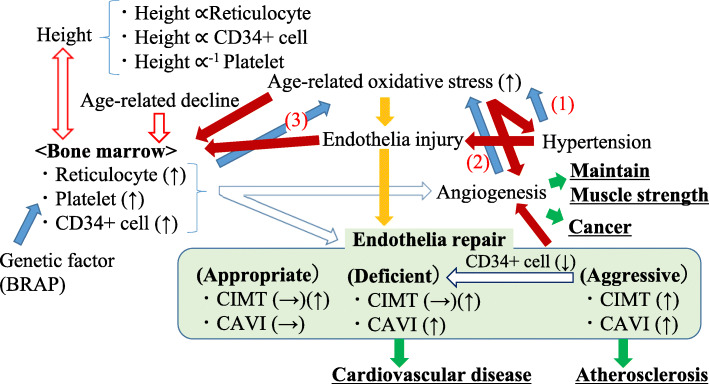
Summary of potential mechanism underlying endothelial maintenance. CAVI, cardio-ankle vascular stiffness index; CIMT, carotid intima-media thickness. The pathway that reduces oxidative stress by hypertension (1), angiogenesis (2), and production of anti-oxidative agents (3)

A summary of the potential mechanisms underlying the endothelial maintenance activity in relation to height is shown in Fig. 5.

Bone marrow activity plays a crucial role in endothelial maintenance by enhancing the production of reticulocytes [56], platelets [90, 96], and CD34-positive cells [15, 26–28, 36, 37, 51]. However, age-related decline reduces the productivity of these cells. Height could influence this age-related decline [29, 52, 53, 67, 91], while BRAP stimulates platelet production [31, 96]. A living body may have three major ways to overcome increased oxidative stress. The first mechanism compensates for blood flow by using the vascular network, which is known as hypertension (Fig. 5(1)). The second one is compensating for blood flow by creating vascular architecture known as angiogenesis (Fig. 5(2)). The last one is increasing the expression levels of the antioxidant (Fig. 5(3)). Reticulocytes have anti-oxidative effects. If enough angiogenesis is established, hypertension is no longer necessary [27], and if enough anti-oxidative effect is established, atherosclerosis is no longer necessary [56]. Active development of angiogenesis could be a crucial risk factor for developing cancer [14], but also has a beneficial influence on muscle strength maintenance [32, 103–105].

Endothelial repair can also be divided into three main categories [26, 28, 51]: appropriate, deficiency, and aggressive. When appropriate endothelial repair is established, even if the organic value of arterial stiffness (CIMT) is elevated, the functional value of arterial stiffness (CAVI) is under control, while aggressive repair elevates both the organic and functional values of arterial stiffness [28]. Even deficiency of endothelial repair increases functional arterial stiffness, organic arterial stiffness may not be increased. In addition, aggressive endothelial repair induces a reduction in CD34-positive cells [26, 28, 29, 36, 37, 90]. Reduction of CD34-positive cells results in comparatively deficient endothelial repair [26]. In this case, both the organic and functional values of arterial stiffness could be elevated.

### Perspective

In an aged society, the disadvantage of an endothelial repair deficiency that is associated with a lack of progression of atherosclerosis could be enhanced. Since the presence of CD34-positive cells is mandatory not only in the progression of atherosclerotic lesions [16, 26] but also to induce angiogenesis involved in the maintenance of microcirculation [38, 39], development of atherosclerosis could indicate sufficient adaptability for increased oxidative stress, especially among the elderly. All reactions occurring in living creatures are programmed to perform beneficial activities for life. Since the progression of atherosclerosis and hypertension are reaction against increased oxidative stress, these should have beneficial effects. Appropriate endothelial maintenance plays an important role in regulating microcirculation [27, 32, 104, 105].

Furthermore, from an anthropological point of view, an extensive prevalence of SNPs should have the same beneficial effect on the daily activities of the participants, rather than imposing a disadvantage. Since genetic characteristics that stimulate progression of atherosclerosis [92, 93] have been revealed to reduce the risk of hypertension [31, 94], the reason why human acquired genetic risk results in the progression of atherosclerosis can be explained by our recent studies.

However, only limited studies have been conducted along with the current concept. There are many unknown factors that take part in the present mechanism. One of these is the genetic factor that relates to height and cardiovascular disease [106]. Furthermore, human height genes have been reported to be associated with cancer [107]. In addition, thyroid hormones are also known to be associated with growth rate [108]. Thyroid hormones regulate bone marrow-derived hematopoietic stem cells [109]. In our previous study, the absence of thyroid cysts was shown to be associated with latent thyroid damage [110–112]. Therefore, thyroid hormones and thyroid cysts could also influence the endothelial maintenance mechanism that relates to different height levels. More elaborate investigations on this concept are strongly desired.

## Conclusion

In this review, the potential mechanism underlying endothelial maintenance by focusing on bone marrow activity is shown. Height, which is an easily measurable factor, has been shown to be associated with bone marrow activity that determines the capacity of endothelial maintenance. Additionally, the beneficial influence of developing atherosclerosis was also shown, which also has a critical influence on the outcome of cardiovascular disease.

## Data Availability

Not applicable

## Abbreviation

CIMT: Carotid intima-media thickness
CAVI: Cardio-ankle vascular index
ISHAGE: International society of hematotherapy and graft engineering
γ-GTP: Gamma-glutamyl transpeptidase
NO: Nitric oxide
BMI: Body mass index
SNP: Single nucleotide polymorphism
BRAP: Breast cancer suppressor BRCA1-related associated protein
ALDH2: Aldehyde dehydrogenase 2
LD: Linkage disequilibrium

## References

[1] Sohn K. The association between height and hypertension in Indonesia. Econ Hum Biol. 2017;27(Pt A):74–83. 10.1016/j.ehb.2017.04.007 28550808

[2] Song L, Shen L, Li H, Liu B, Zheng X, Liang Y (2016). Height and prevalence of hypertension in a middle-aged and older Chinese population. Sci Rep.

[3] Sawada N, Wark PA, Merritt MA, Tsugane S, Ward HA, Rinaldi S, et al. The association between adult attained height and sitting height with mortality in the European Prospective Investigation into Cancer and Nutrition (EPIC). PLoS One. 2017;12(3):e0173117. 10.1371/journal.pone.0173117 PMC5336260 28257491

[4] Hozawa A, Murakami Y, Okamura T, Kadowaki T, Nakamura K, Hayakawa T (2007). NIPPON DATA80 Research Group. Relation of adult height with stroke mortality in Japan: NIPPON DATA80. Stroke.

[5] Honjo K, Iso H, Inoue M, Tsugane S (2011). Adult height and the risk of cardiovascular disease among middle aged men and women in Japan. Eur J Epidemiol.

[6] Park CS, Choi EK, Han KD, Lee HJ, Rhee TM, Lee SR (2018). Association between adult height, myocardial infarction, heart failure, stroke and death: a Korean nationwide population-based study. Int J Epidemiol.

[7] Shimizu Y, Imano H, Ohira T, Kitamura A, Kiyama M, Okada T, et al. CIRCS Investigators. Adult height and body mass index in relation to risk of total stroke and its subtypes: the circulatory risk in communities study. J Stroke Cerebrovasc Dis. 2014;23(4):667–74. 10.1016/j.jstrokecerebrovasdis.2013.06.009 23871699

[8] Ong JS, An J, Law MH, Whiteman DC, Neale RE, Gharahkhani P, et al. Height and overall cancer risk and mortality: evidence from a Mendelian randomisation study on 310,000 UK biobank participants. Br J Cancer. 2018;118(9):1262–7. 10.1038/s41416-018-0063-4 PMC5943400 29581483

[9] Salminen A, Kaarniranta K, Kauppinen A. Hypoxia-inducible histone lysine demethylases: Impact on the aging process and age-related diseases. Aging Dis. 2016;7(2):180–200. 10.14336/AD.2015.0929 PMC4809609 27114850

[10] Eltzschig HK, Carmeliet P (2011). Hypoxia and inflammation. N Engl J Med.

[11] García N, Zazueta C, Aguilera-Aguirre L (2017). Oxidative stress and inflammation in cardiovascular disease. Oxid Med Cell Longev.

[12] Klaunig JE (2018). Oxidative stress and cancer. Curr Pharm Des.

[13] Murata M (2018). Inflammation and cancer. Environ Health Prev Med.

[14] Viallard C, Larrivée B (2017). Tumor angiogenesis and vascular normalization: alternative therapeutic targets. Angiogenesis.

[15] Shi Q, Rafii S, Wu MH, Wijelath ES, Yu C, Ishida A (1998). Evidence for circulating bone marrow-derived endothelial cells. Blood.

[16] Daub K, Langer H, Seizer P, Stellos K, May AE, Goyal P (2006). Platelets induce differentiation of human CD34+ progenitor cells into foam cells and endothelial cells. FASEB J.

[17] Guralnik JM, Ershler WB, Schrier SL, Picozzi VJ. Anemia in the elderly: a public health crisis in hematology. Hematology Am Soc Hematol Educ Program. 2005:528–32. 10.1182/asheducation-2005.1.528 16304431

[18] Cooper B (2011). The origins of bone marrow as the seedbed of our blood: from antiquity to the time of Osler. Proc (Bayl Univ Med Cent).

[19] National Institute of Population and Social Security Research [Home page on the Internet]. Available at [Cited February 1. 2021]: http://www.ipss.go.jp/pp-shicyoson/j/shicyoson13/5fusa/Municipalities.asp.

[20] Sutherland DR, Anderson L, Keeney M, Nayar R, Chin-Yee I (1996). The ISHAGE guidelines for CD34+ cell determination by flow cytometry. International Society of Hematotherapy and Graft Engineering. J Hematother.

[21] Hara T, Takamura N, Akashi S, Nakazato M, Maeda T, Wada M (2006). Evaluation of clinical markers of atherosclerosis in young and elderly Japanese adults. Clin Chem Lab Med.

[22] Yanase T, Nasu S, Mukuta Y, Shimizu Y, Nishihara T, Okabe T (2006). Evaluation of a new carotid intima-media thickness measurement by B-mode ultrasonography using an innovative measurement software, intimascope. Am J Hypertens.

[23] Yamashina A, Tomiyama H, Arai T, Koji Y, Yambe M, Motobe H (2003). Nomogram of the relation of brachial-ankle pulse wave velocity with blood pressure. Hypertens Res.

[24] Shirai K, Song M, Suzuki J, Kurosu T, Oyama T, Nagayama D (2011). Contradictory effects of β1- and α1- aderenergic receptor blockers on cardio-ankle vascular stiffness index (CAVI)--CAVI independent of blood pressure. J Atheroscler Thromb.

[25] Yambe T, Yoshizawa M, Saijo Y, Yamaguchi T, Shibata M, Konno S (2004). Bracho-ankle pulse wave velocity and cardio-ankle vascular index (CAVI). Biomed Pharmacother.

[26] Shimizu Y, Kawashiri SY, Kiyoura K, Koyamatsu J, Fukui S, Tamai M, et al. Circulating CD34+ cells and active arterial wall thickening among elderly men: A prospective study. Sci Rep. 2020;10(1):4656. 10.1038/s41598-020-61475-4 PMC7069955 32170211

[27] Shimizu Y, Kawashiri SY, Kiyoura K, Nobusue K, Yamanashi H, Nagata Y (2019). Gamma-glutamyl transpeptidase (γ-GTP) has an ambivalent association with hypertension and atherosclerosis among elderly Japanese men: a cross-sectional study. Environ Health Prev Med.

[28] Shimizu Y, Yamanashi H, Noguchi Y, Koyamatsu J, Nagayoshi M, Kiyoura K, et al. Cardio-ankle vascular index and circulating CD34-positive cell levels as indicators of endothelial repair activity in older Japanese men. Geriatr Gerontol Int. 2019;19(6):557–62. 10.1111/ggi.13657 30920121

[29] Shimizu Y, Yamanashi H, Noguchi Y, Koyamatsu J, Nagayoshi M, Kiyoura K (2019). Association between height and circulating CD34-positive cells taken into account for the influence of enhanced production among elderly Japanese men: a cross-sectional study. Aging (Albany NY).

[30] Shimizu Y, Nakazato M, Sekita T, Kadota K, Arima K, Yamasaki H (2013). Relationship between adult height and body weight and risk of carotid atherosclerosis assessed in terms of carotid intima-media thickness: the Nagasaki Islands study. J Physiol Anthropol..

[31] Shimizu Y, Arima K, Noguchi Y, Kawashiri SY, Yamanashi H, Tamai M (2020). Potential mechanisms underlying the association between single nucleotide polymorphism (BRAP and ALDH2) and hypertension among elderly Japanese population. Sci Rep.

[32] Shimizu Y, Sato S, Koyamatsu J, Yamanashi H, Nagayoshi M, Kadota K (2017). Handgrip strength and subclinical carotid atherosclerosis in relation to platelet levels among hypertensive elderly Japanese. Oncotarget.

[33] Bots ML, Grobbee DE (2002). Intima media thickness as a surrogate marker for generalised atherosclerosis. Cardiovasc Drugs Ther.

[34] van den Oord SC, Sijbrands EJ, ten Kate GL, van Klaveren D, van Domburg RT, van der Steen AF (2013). Carotid intima-media thickness for cardiovascular risk assessment: systematic review and meta-analysis. Atherosclerosis.

[35] Lorenz MW, Polak JF, Kavousi M, Mathiesen EB, Völzke H, Tuomainen TP (2012). PROG-IMT Study Group. Carotid intima-media thickness progression to predict cardiovascular events in the general population (the PROG-IMT collaborative project): a meta-analysis of individual participant data. Lancet.

[36] Shimizu Y, Sato S, Koyamatsu J, Yamanashi H, Nagayoshi M, Kadota K, et al. Platelets and circulating CD34-positive cells as an indicator of the activity of the vicious cycle between hypertension and endothelial dysfunction in elderly Japanese men. Atherosclerosis. 2017;259:26–31. 10.1016/j.atherosclerosis.2017.02.016 28282559

[37] Shimizu Y, Sato S, Koyamatsu J, Yamanashi H, Nagayoshi M, Kadota K (2015). Circulating CD34-positive cells, glomerular filtration rate and triglycerides in relation to hypertension. Atherosclerosis.

[38] Siemerink MJ, Klaassen I, Vogels IM, Griffioen AW, Van Noorden CJ, Schlingemann RO. CD34 marks angiogenic tip cells in human vascular endothelial cell cultures. Angiogenesis. 2012;15(1):151–63. 10.1007/s10456-011-9251-z PMC3274677 22249946

[39] Takakura N, Watanabe T, Suenobu S, Yamada Y, Noda T, Ito Y (2000). A role for hematopoietic stem cells in promoting angiogenesis. Cell.

[40] Kattoor AJ, Pothineni NVK, Palagiri D, Mehta JL. Oxidative stress in atherosclerosis. Curr Atheroscler Rep. 2017;19(11):42. 10.1007/s11883-017-0678-6 28921056

[41] Baradaran A, Nasri H, Rafieian-Kopaei M. Oxidative stress and hypertension: Possibility of hypertension therapy with antioxidants. J Res Med Sci. 2014;19(4):358–67. PMC4115353 25097610

[42] Cho AR, Kwon YJ, Lim HJ, Lee HS, Kim S, Shim JY, et al. Oxidative balance score and serum γ-glutamyltransferase level among Korean adults: a nationwide population-based study. Eur J Nutr. 2018;57(3):1237–44. 10.1007/s00394-017-1407-1 28258305

[43] Uçar H, Gür M, Gözükara MY, Kalkan GY, Baykan AO, Türkoğlu C (2015). Gamma glutamyl transferase activity is independently associated with oxidative stress rather than SYNTAX score. Scand J Clin Lab Invest.

[44] Liochev SI (2015). Reflections on the theories of aging, of oxidative stress, and of science in general. Is it time to abandon the free radical (oxidative stress) theory of aging?. Antioxid Redox Signal.

[45] Satoh T, Yamakage M, Satoh J, Namiki A (2007). Effect of aging on preoperative oxidative stress. Masui.

[46] Yeo EJ (2019). Hypoxia and aging. Exp Mol Med.

[47] Irarrázaval S, Allard C, Campodónico J, Pérez D, Strobel P, Vásquez L (2017). Oxidative stress in acute hypobaric hypoxia. High Alt Med Biol.

[48] McGarry T, Biniecka M, Veale DJ, Fearon U (2018). Hypoxia, oxidative stress and inflammation. Free Radic Biol Med.

[49] Takaki A, Ogawa H, Wakeyama T, Iwami T, Kimura M, Hadano Y (2007). Cardio-ankle vascular index is a new noninvasive parameter of arterial stiffness. Circ J.

[50] Shimizu M, Furusyo N, Mitsumoto F, Takayama K, Ura K, Hiramine S (2015). Subclinical carotid atherosclerosis and triglycerides predict the incidence of chronic kidney disease in the Japanese general population: results from the Kyushu and Okinawa Population Study (KOPS). Atherosclerosis.

[51] Shimizu Y, Yamanashi H, Noguchi Y, Koyamatsu J, Nagayoshi M, Kiyoura K, et al. Association between chronic kidney disease and carotid intima-media thickness in relation to circulating CD34-positive cell count among community-dwelling elderly Japanese men. Atherosclerosis. 2019;283:85–91. 10.1016/j.atherosclerosis.2019.02.004 30818167

[52] Shimizu Y, Sato S, Koyamatsu J, Yamanashi H, Nagayoshi M, Kadota K, et al. Height indicates hematopoietic capacity in elderly Japanese men. Aging (Albany NY). 2016;8(10):2407–13. 10.18632/aging.101061 PMC5115896 27705902

[53] Shimizu Y, Nakazato M, Sekita T, Kadota K, Miura Y, Arima K (2015). Height and drinking status in relation to risk of anemia in rural adult healthy Japanese men: the Nagasaki Islands study. Aging Male.

[54] Malenganisho W, Magnussen P, Vennervald BJ, Krarup H, Kaestel P, Siza J (2007). Intake of alcoholic beverages is a predictor of iron status and hemoglobin in adult Tanzanians. J Nutr.

[55] Milman N, Pedersen AN (2009). Blood haemoglobin concentrations are higher in smokers and heavy alcohol consumers than in non-smokers and abstainers: should we adjust the reference range?. Ann Hematol.

[56] Shimizu Y, Kawashiri SY, Yamanashi H, Koyamatsu J, Fukui S, Kondo H, et al. Reticulocyte levels have an ambivalent association with hypertension and atherosclerosis in the elderly: a cross-sectional study. Clin Interv Aging. 2019;14:849–57. 10.2147/CIA.S197982 PMC6512840 31190771

[57] Shimizu Y, Sato S, Koyamatsu J, Yamanashi H, Nagayoshi M, Kadota K, et al. Possible mechanism underlying the association between higher hemoglobin level and hypertension in older Japanese men. Geriatr Gerontol Int. 2017;17(12):2586–92. 10.1111/ggi.13068 28581690

[58] Shimizu Y, Nakazato M, Sekita T, Kadota K, Arima K, Yamasaki H (2014). Association between the hemoglobin levels and hypertension in relation to the BMI status in a rural Japanese population: the Nagasaki Islands Study. Intern Med.

[59] Shimizu Y, Kadota K, Nakazato M, Noguchi Y, Koyamatsu J, Yamanashi H (2016). Hemoglobin as a possible biochemical index of hypertension-induced vascular damage. J Physiol Anthropol.

[60] VaSera operation manual, Fukuda Denshi CO.,LTD, Tokyo, Japan. Available at [Cited February 1. 2021]. http://www.fukuda.co.jp/english/products/special_features/vasera/cavi.html.

[61] Shimizu Y, Nakazato M, Sekita T, Kadota K, Yamasaki H, Takamura N, et al. Association between hemoglobin levels and arterial stiffness for general Japanese population in relation to body mass index status: The Nagasaki Islands study. Geriatr Gerontol Int. 2014;14(4):811–8. 10.1111/ggi.12171 24215101

[62] Grillo A, Salvi L, Coruzzi P, Salvi P, Parati G (2019). Sodium intake and hypertension. Nutrients.

[63] Pilic L, Pedlar CR, Mavrommatis Y (2016). Salt-sensitive hypertension: mechanisms and effects of dietary and other lifestyle factors. Nutr Rev.

[64] Terpolilli NA, Feiler S, Dienel A, Müller F, Heumos N, Friedrich B (2016). Nitric oxide inhalation reduces brain damage, prevents mortality, and improves neurological outcome after subarachnoid hemorrhage by resolving early pial microvasospasms. J Cereb Blood Flow Metab.

[65] Bragulat E, de la Sierra A (2002). Salt intake, endothelial dysfunction, and salt-sensitive hypertension. J Clin Hypertens (Greenwich).

[66] Wonisch W, Falk A, Sundl I, Winklhofer-Roob BM, Lindschinger M. Oxidative stress increases continuously with BMI and age with unfavourable profiles in males. Aging Male. 2012;15(3):159–65. 10.3109/13685538.2012.669436 22468695

[67] Shimizu Y, Sato S, Koyamatsu J, Yamanashi H, Nagayoshi M, Kadota K (2017). Height is an indicator of vascular maintenance capacity in older men. Geriatr Gerontol Int.

[68] Kitamura A, Yamagishi K, Imano H, Kiyama M, Cui R, Ohira T, et al. CIRCS Investigators. Impact of hypertension and subclinical organ damage on the incidence of cardiovascular disease among Japanese residents at the population and individual levels – The Circulatory Risk in Communities Study (CIRCS). Circ J. 2017;81(7):1022–8. 10.1253/circj.CJ-16-1129 28367846

[69] Curb JD, Marcus EB (1991). Body fat and obesity in Japanese Americans. Am J Clin Nutr.

[70] Curb JD, Marcus EB. Body fat, coronary heart disease, and stroke in Japanese men. Am J Clin Nutr. 1991;53(6 Suppl):1612–5. 10.1093/ajcn/53.6.1612S 2031494

[71] Mitsuyama Y, Thompson LR, Hayashi T, Lee KK, Keehn RJ, Resch JA (1979). Autopsy study of cerebrovascular disease in Japanese men who lived in Hiroshima, Japan, and Honolulu, Hawaii. Stroke.

[72] Pleşea IE, Cameniţă A, Georgescu CC, Enache SD, Zaharia B, Georgescu CV, et al. Study of cerebral vascular structures in hypertensive intracerebral haemorrhage. Rom J Morphol Embryol. 2005;46(3):249–56. 16444313

[73] Taguchi A, Matsuyama T, Moriwaki H, Hayashi T, Hayashida K, Nagatsuka K, et al. Circulating CD34-positive cells provide an index of cerebrovascular function. Circulation. 2004;109(24):2972–5. 10.1161/01.CIR.0000133311.25587.DE 15184275

[74] Shimizu Y, Yoshimine H, Nagayoshi M, Kadota K, Takahashi K, Izumino K (2016). Height correlates with dyslipidemia in non-overweight middle-aged Japanese men. J Physiol Anthropol.

[75] WHO Expert Consultation. Appropriate body-mass index for Asian populations and its implications for policy and intervention strategies. Lancet. 2004;363(9403):157–63. 10.1016/S0140-6736(03)15268-3 14726171

[76] Shimizu Y, Yoshimine H, Nagayoshi M, Kadota K, Takahashi K, Izumino K (2016). Short stature is an inflammatory disadvantage among middle-aged Japanese men. Environ Health Prev Med.

[77] Sakata K, Labarthe DR (1996). Changes in cardiovascular disease risk factors in three Japanese national surveys 1971-1990. J Epidemiol.

[78] Ikeda N, Gakidou E, Hasegawa T, Murray CJ (2008). Understanding the decline of mean systolic blood pressure in Japan: an analysis of pooled data from the National Nutrition Survey, 1986-2002. Bull World Health Organ.

[79] Hata J, Ninomiya T, Hirakawa Y, Nagata M, Mukai N, Gotoh S (2013). Secular trends in cardiovascular disease and its risk factors in Japanese: half-century data from the Hisayama Study (1961-2009). Circulation.

[80] Funatogawa I, Funatogawa T, Nakao M, Karita K, Yano E. Changes in body mass index by birth cohort in Japanese adults: results from the National Nutrition Survey of Japan 1956-2005. Int J Epidemiol. 2009;38(1):83–92. 10.1093/ije/dyn182 PMC2639362 18782894

[81] Kouchi M. Secular change and socioeconomic difference in height in Japan. Anthropol Sci. 1996;104(4):325–40.

[82] Cao J, Eshak ES, Liu K, Gero K, Liu Z, Yu C. Age-period-cohort analysis of stroke mortality attributable to high sodium intake in China and Japan. Stroke. 2019;50(7):1648–54. 10.1161/STROKEAHA.118.024617 PMC6594775 31195942

[83] Tarui I, Okada E, Okada C, Saito A, Takimoto H (2020). Trends in BMI among elderly Japanese population: findings from 1973 to 2016 Japan National Health and Nutrition Survey. Public Health Nutr.

[84] Lindemann S, Krämer B, Seizer P, Gawaz M (2007). Platelets, inflammation and atherosclerosis. J Thromb Haemost.

[85] Report of a meeting of physicians and scientists, University of Texas Health Science Center at Houston and Texas Heart Institute, Houston. Platelet activation and arterial thrombosis. Lancet. 1994;344(8928):991–5. 7934435

[86] Nakamura T, Kambayashi J, Okuma M, Tandon NN. Activation of the GP IIb-IIIa complex induced by platelet adhesion to collagen is mediated by both alpha2beta1 integrin and GP VI. J Biol Chem. 1999;274(17):11897–903. 10.1074/jbc.274.17.11897 10207010

[87] Stellos K, Langer H, Daub K, Schoenberger T, Gauss A, Geisler T (2008). Platelet-derived stromal cell-derived factor-1 regulates adhesion and promotes differentiation of human CD34+ cells to endothelial progenitor cells. Circulation.

[88] Stellos K, Bigalke B, Langer H, Geisler T, Schad A, Kögel A (2009). Expression of stromal-cell-derived factor-1 on circulating platelets is increased in patients with acute coronary syndrome and correlates with the number of CD34+ progenitor cells. Eur Heart J.

[89] Seitz G, Boehmler AM, Kanz L, Möhle R. The role of sphingosine 1-phosphate receptors in the trafficking of hematopoietic progenitor cells. Ann N Y Acad Sci. 2005;1044:84–9. 10.1196/annals.1349.011 15958700

[90] Shimizu Y, Sato S, Koyamatsu J, Yamanashi H, Nagayoshi M, Kadota K, et al. Platelets as an indicator of vascular repair in elderly Japanese men. Oncotarget. 2016;7(29):44919–26. 10.18632/oncotarget.10229 PMC5216694 27374094

[91] Shimizu Y, Sato S, Koyamatsu J, Yamanashi H, Nagayoshi M, Kadota K (2017). Possible mechanism underlying the association between height and vascular remodeling in elderly Japanese men. Oncotarget.

[92] Ozaki K, Sato H, Inoue K, Tsunoda T, Sakata Y, Mizuno H, et al. SNPs in BRAP associated with risk of myocardial infarction in Asian populations. Nat Genet. 2009;41(3):329–33. 10.1038/ng.326 19198608

[93] Liao YC, Wang YS, Guo YC, Ozaki K, Tanaka T, Lin HF (2011). BRAP activates inflammatory cascades and increases the risk for carotid atherosclerosis. Mol Med.

[94] Yamada Y, Sakuma J, Takeuchi I, Yasukochi Y, Kato K, Oguri M (2017). Identification of polymorphisms in 12q24.1, ACAD10, and BRAP as novel genetic determinants of blood pressure in Japanese by exome-wide association studies. Oncotarget.

[95] Liao YC, Lin HF, Guo YC, Chen CH, Huang ZZ, Juo SH (2013). Lack of association between a functional variant of the BRCA-1 related associated protein (BRAP) gene and ischemic stroke. BMC Med Genet.

[96] Shimizu Y, Yamanashi H, Noguchi Y, Koyamatsu J, Nagayoshi M, Kiyoura K (2019). Short stature-related single-nucleotide polymorphism (SNP) activates endothelial repair activity in elderly Japanese. Environ Health Prev Med.

[97] Matsumoto A (2019). The bidirectional effect of defective ALDH2 polymorphism and disease prevention. Adv Exp Med Biol.

[98] Kamatani Y, Matsuda K, Okada Y, Kubo M, Hosono N, Daigo Y (2010). Genome-wide association study of hematological and biochemical traits in a Japanese population. Nat Genet.

[99] Kim JW, Choe YM, Shin JG, Park BL, Shin HD, Choi IG, et al. Associations of BRAP polymorphisms with the risk of alcohol dependence and scores on the alcohol use disorders identification test. Neuropsychiatr Dis Treat. 2018;15:83–94. 10.2147/NDT.S184067 PMC6309135 30636874

[100] Stach K, Kälsch AI, Weiß C, Elmas E, Borggrefe M, Kälsch T (2012). Effects of ethanol on the properties of platelets and endothelial cells in model experiments. World J Cardiol.

[101] Kim GR, Sun J, Han M, Park S, Nam CM. Impact of handgrip strength on cardiovascular, cancer and all-cause mortality in the Korean longitudinal study of ageing. BMJ Open. 2019;9(5):e027019. 10.1136/bmjopen-2018-027019 PMC6527975 31072857

[102] Xue Q, Qin MZ, Jia J, Liu JP, Wang Y (2019). Association between frailty and the cardio-ankle vascular index. Clin Interv Aging.

[103] Torii K, Shimizu Y, Sato S, Noguchi Y, Koyamatsu J, Yamanashi H (2018). Reduced tongue pressure and platelet count in relation to hypertension among community dwelling elderly Japanese subjects. Acta Medica Nagasakiensia.

[104] Taekema DG, Maier AB, Westendorp RG, de Craen AJ (2011). Higher blood pressure is associated with higher handgrip strength in the oldest old. Am J Hypertens.

[105] Shimizu Y, Sato S, Noguchi Y, Koyamatsu J, Yamanashi H, Higashi M (2018). Association between tongue pressure and subclinical carotid atherosclerosis in relation to platelet levels in hypertensive elderly men: a cross-sectional study. Environ Health Prev Med.

[106] Nelson CP, Hamby SE, Saleheen D, Hopewell JC, Zeng L, Assimes TL, et al. CARDIoGRAM+C4D Consortium. Genetically determined height and coronary artery disease. N Engl J Med. 2015;372(17):1608–18. 10.1056/NEJMoa1404881 PMC4648271 25853659

[107] Tripaldi R, Stuppia L, Alberti S (2013). Human height genes and cancer. Biochim Biophys Acta.

[108] Tarım Ö (2011). Thyroid hormones and growth in health and disease. J Clin Res Pediatr Endocrinol.

[109] Mogharbel BF, Abdelwahid E, Irioda AC, Francisco JC, Simeoni RB, de Souza D (2017). Bone marrow-derived stem cell populations are differentially regulated by thyroid or/and ovarian hormone loss. Int J Mol Sci.

[110] Shimizu Y, Kawashiri SY, Noguchi Y, Nagata Y, Maeda T, Hayashida N (2020). Anti-thyroid peroxidase antibody and subclinical hypothyroidism in relation to hypertension and thyroid cysts. PLoS One.

[111] Shimizu Y, Nabeshima-Kimura Y, Kawashiri SY, Noguchi Y, Nagata Y, Maeda T, et al. Anti-thyroid peroxidase antibody and thyroid cysts among the general Japanese population: a cross-sectional study. Environ Health Prev Med. 2020;25(1):7. 10.1186/s12199-020-00844-x PMC7035657 32085700

[112] Shimizu Y, Nabeshima-Kimura Y, Kawashiri SY, Noguchi Y, Nagata Y, Maeda T (2020). Associations between thyroid-stimulating hormone and hypertension according to thyroid cyst status in the general population: a cross-sectional study. Environ Health Prev Med.

